# A Molecular Dynamics Simulations Study of the Influence of Prestrain on the Pop-In Behavior and Indentation Size Effect in Cu Single Crystals

**DOI:** 10.3390/ma14185220

**Published:** 2021-09-10

**Authors:** Rong-Guang Xu, Hengxu Song, Yongsheng Leng, Stefanos Papanikolaou

**Affiliations:** 1 Department of Mechanical and Aerospace Engineering, The George Washington University, Washington, DC 20052, USA; leng@gwu.edu; 2 Institute for Advanced Simulations—Materials Data Science and Informatics (IAS-9), Forschungszentrum Jülich GmbH, 52425 Jülich, Germany; songhengxu@gmail.com; 3 NOMATEN Centre of Excellence, National Centre of Nuclear Research, A. Soltana 7, 05-400 Otwock, Poland

**Keywords:** nanoindentation, avalanches, pop-ins

## Abstract

The pop-in effect in nanoindentation of metals represents a major collective dislocation phenomenon that displays sensitivity in the local surface microstructure and residual stresses. To understand the deformation mechanisms behind pop-ins in metals, large scale molecular dynamics simulations are performed to investigate the pop-in behavior and indentation size effect in undeformed and deformed Cu single crystals. Tensile loading, unloading, and reloading simulations are performed to create a series of samples subjected to a broad range of tensile strains with/without pre-existing dislocations. The subsequent nanoindentation simulations are conducted to investigate the coupled effects of prestrain and the presence of resulting dislocations and surface morphology, as well as indenter size effects on the mechanical response in indentation processes. Our work provides detailed insights into the deformation mechanisms and microstructure-property relationships of nanoindentation in the presence of residual stresses and strains.

## 1. Introduction

Nanoindentation is a widely used technique to investigate mechanical responses of small volumes of materials at micro- and nano-length scales [1]. Indentation size effect (ISE), i.e., the hardness increases with the decreasing indentation depth (for sharp indenters) or with the decreasing indenter radius (for spherical indenters), opens up a new direction for the study of metal plasticity. The pioneering theoretical work dated back to 1998, when Nix and Gao explained ISE by utilizing the concept of geometrically necessary dislocations (GND) to construct a mesoscale theory of strain-gradient plasticity based on the model of Taylor hardening [2]. One of the typical features of the plasticity at the small length scale is that the mechanical response (stress strain curve in uniaxial compression of pillar or force depth curve in nanoindentation) is intermittent. The intermittency is termed as pop-in in nanoindentation, which is a displacement burst [3] in a load-controlled loading system or a force drop in a displacement-controlled loading system. The first pop-in event manifests the onset of plasticity and is believed to correspond to the nucleation of dislocations in a confined dislocation-free volume; therefore, the shear strength when pop-in events occur is thought to be close to the theoretical strength G/2π, where G is the shear modulus. However, it is found that as the indenter radius and prestrain of the sample decrease, the first pop-in loads decrease and increase, respectively, and the resulting maximum shear stresses derived from the pop-in loads increase [3,4,5]. This phenomenon exhibits another type of ISE: the stress required to initiate dislocation plasticity also depends on the size.

For the study of the crystal plasticity at the small length scale, molecular dynamics (MD) simulation has significant advantages: it can provide an atomistic picture of the loading process, offering many details of dislocation generation, propagation, reaction, and annihilation [6,7,8]. MD simulations were successfully performed and made tremendous contributions in understanding various aspects of nanoindentation. However, most of the previous MD studies were based on perfect crystal, in which no pre-existing dislocations existed in the simulation systems. In reality, dislocations cannot be ignored since even for well-annealed metals, the dislocation density is in the range of 10^6^–10^8^ cm/cm^3^_._ At the nanoscale, the role of existing dislocations is even more prominent, therefore, a defect-based model is needed to explain the real nanoindentation experiments.

The effects of a variety of pre-existing defects such as vacancies, self-interstitial atoms and stacking fault tetrahedra (SFTs) on the incipient plasticity in dislocation-free metals were reported [9,10]. The critical load decreases due to the presence of such defects. In a recent MD study [11], the indentation on a CaF_2_ perfect crystal were performed in two steps. The first indentation was performed using a large indenter (R = 12 nm) to induce plastic deformation, and this is then followed by a second indentation with a small indenter (R = 4 nm) performed in the middle of the predeformed zone. The interactions of pre-existing dislocations with the newly nucleated dislocations through the second indentation were studied. A smooth transition from elasticity to plasticity without a significant force drop was observed. In a similar study [12], plastic deformation was induced by nano-scratching with a 4 nm spherical tip, then the nanoindentation with a 3 nm spherical tip was carried out to assess the subsurface damage. Their simulations reveal that the maximum hardness decreases continuously with increasing machining depth of the surface, while the indentation hardness has no strong dependence on the prior nanomachining. In a more recent MD simulation [13], single-, two-, and three-extended edge dislocations (EEDs) were introduced by removing one or more sets of two half layers of atoms. Subsequent MD simulations of nanoindentation investigated the mechanism of incipient plasticity in terms of the mutual interaction of the existing dislocations. A newly published paper investigated the influence of pre-existing defects on hardness in nanoscale indentations, in which misfit dislocations was nucleated at semicoherent Ti/Al bicrystal interface [14].

Cu is one of the most widely used metal materials studied in the nanoindentation experiment [15] and simulations [9,10,16,17]. The topic is so broad that any attempt to perform an exhaustive review in the domain may easily overlook some important work in the field. To name a few, Li et al. [7] carried out MD simulations combined with finite-element modeling and experimental analysis to quantify the atomistic mechanism of defect nucleation and incipient plasticity in nanoindentation of single crystalline Cu and Al. The effect of Cu’s anisotropy on elastic and plastic deformation in nanoindetation was studied by MD simulations [18]. For the case of MD simulations of nanocrystalline Cu, Ma and Yang found that in contrast to single crystal Cu, the burst and arrest of stacking faults account for the plastic deformation of nanocrystalline sample under nanoindentation [19]. A recent simulation work more relevant to our research investigated the nanoindentation response of Cu under elastic tensile strain [20]. Besides indentation simulations, the mechanical behaviors of prestrained Cu thin films were also investigated by MD simulations [21,22].

In this study, we introduce the dislocations in nanoindentation samples through a very natural manner by uniaxial straining a face-centered cubic (FCC) single crystal Cu to the plastic regime. This approach follows our recent nanoindentation experiments that showed concrete effects of in-situ tensile loads on pop-in noise in Cu single crystals for depths up to 50 nm [23]. Subsequent MD simulations of indentation are carried out to explore the coupled effects of prestrain, the presence of the resulting dislocations, and the indenter size on the nanoindentation response of the Cu single crystal.

The remainder of this paper is organized as follows. In Section 2, the materials and simulation methods are described in detail. MD simulation results are presented in Section 3, followed by conclusions in Section 4.

## 2. Materials and Methods

**Figure 1:**
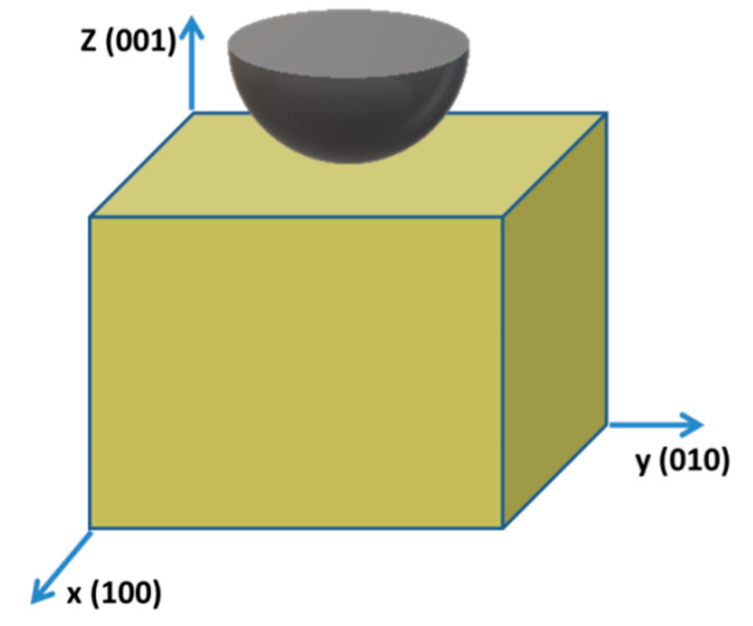
Schematic representation of nanoindentation simulations. Indentations performed in (001) direction. Virtual indenter is represented by a nonatomistic rigid sphere. Nanoindentation spot is at (x = 10 nm, y = 10 nm).

A substrate made of FCC single crystal Cu was initially constructed with $20\times 20\times 20{\;\mathrm{nm}}^{3}$ in size and containing 702,464 Cu atoms, which is similar to previous studies [9,10,12,17]. The x, y, and z Cartesian axes are along the [100], [010], and [001] crystallographic directions, respectively (See Figure 1). In this study, we model the interatomic interactions between Cu atoms with well-established embedded atom method (EAM) potential. All the simulations are performed using the LAMMPS code [24].

Periodic boundary conditions (PBC) are implemented along directions (x and y directions) parallel to the indented surface to approximate the effect of one layer in the z direction that extend without surface in the lateral directions. For tensile prestraining, the two (001) planes are free surfaces with zero traction. For indentation simulations, the bottom surface is held fixed to prevent rigid-body motion of the sample, and atoms in the rest part of are free to move. In these simulations, temperature and pressure, along x and y directions, are maintained in equilibrium with a heat bath and mechanical reservoir using a Nosé–Hoover/Parinello–Rahman formalism [25,26,27,28,29]. The temperature of the simulating system is controlled at 10 K to avoid a temperature effect. The time step is 1 fs and the thermostatting/barostatting time scales is 2 ps.

Initially, the substrate is relaxed under NPT ensemble control for 2 ns at 10 K and 0 GPa along x and y directions. Then, the substrate is deformed in the x direction at a constant strain rate of 10^9^ s^−1^ until strain = 0.6 is reached, during which the substrate undergoes elastic and then plastic deformations. Thus, the dislocation distributions are created and stored in the Cu substrate. The subsequent nanoindentation simulations are then conducted on the (001) surface of the system (see Figure 1). The virtual indenter is represented by a nonatomistic rigid sphere with repulsive potential interacting with atoms in the substrate [6]. The form of such potential is adopted as

(1) $$V\left(r\right)=\{\begin{array}{c} \frac{1}{3}k{\left(R-r\right)}^{3},\;r<R\\ 0,\;\;\;\;\;\;\;\;\;\;\;r\geq R \end{array}$$

This setup allows for indenter with larger radius than the indenter with atomistic characteristics, the size of which is limited by periodic boundary conditions. Since the commonly used indenter in MD simulations has the radius ranging from 1 nm [16] to 15 nm [9,10], indenter tips with radius of 1, 4, 7.5, and 15 nm are adopted in this study. The indentation simulations are carried out in a displacement-control mode and the indenter is moving downward with a speed of 2 m/s.

The indentation force F is calculated by the sum of the forces exerted by all atoms in the substrate on the indenter along z direction, i.e., vertical to the top surface. To obtain the variations of the hardness (here defined as the contact pressure) as a function of the indentation depth, it is crucial to calculate contact area properly. We followed the method in Ref. [18], where the elliptic contact area is calculate by $A^{elliptic}=\frac{\pi }{4}\left(x^{max}-x^{min}\right)\left(y^{max}-y^{min}\right)$, i.e., identifying four atoms in contact with the indenter which have the largest positive or negative x and y coordinates. Here, x and y are the coordinates of the contact atoms projected into the initial surface plane. Also, according to the Hertzian solution [30], the maximum elastic shear stress (*τ_max_*) at pop-in can be obtained by $\tau_{max}=0.31p_{max}$, where the maximum contact pressure is

(2) $$p_{max}={\left(\frac{6F{E^{*}}^{2}}{\pi^{3}R^{2}}\right)}^{1/3},$$

where $E^{*}$ is the reduced elastic modulus defined as $E^{*}=E/1-\nu^{2}$.

Finally, the OVITO open visualization tool [31] is used to identify the atom’s local structure by the common neighbor analysis (CNA) [32] and monitor dislocation movement [31].

## 3. Results

### 3.1. Indentations on Samples without Pre-Strain

**Figure 2:**
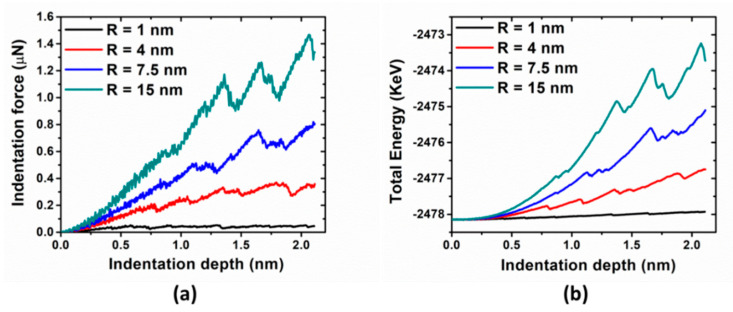
(**a**) Indentation force and (**b**) total energy vs. indentation depth for perfect crystal, indented with different tip radii.

As the first step, nanoindentation simulations are performed on the perfect crystal by four indenters with different tip radii. The force-depth curves are shown in Figure 2a. For all cases, the substrate initially deform elastically until the first force drop. The data before the force drop can be fitted well to the Hertzian solution, i.e., a power-law relationship, F ∞ d^3/2^. The first force drops (also called pop-in load or critical load) are 0.04, 0.21, 0.38, and 0.60 µN for 1, 4, 7.5, and 15 nm indenter tips, respectively, and the corresponding indentation depths are 0.42, 0.77, 0.82, and 0.86 nm. The increase both in the critical force and depth with increase in the indenter tip size is due to the larger stressed volume with the larger tip radius, which is consistent with previous MD simulations [13].

**Figure 3:**
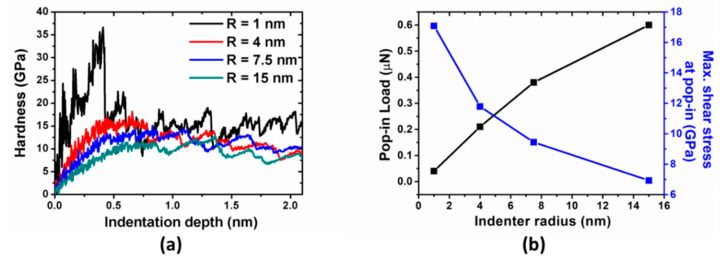
(**a**) Hardness vs. indentation depth with different tip radii; (**b**) pop-in loads (black), and corresponding maximum shear stresses (blue) as a function of indenter radius.

Two types of the indentation size effect can be reproduced by measuring indentation hardness, pop-in load, and corresponding maximum shear stress with different tip radius. Hardness-depth curves are shown in Figure 3a. Initially, the hardness curves increase with d^1/2^-dependence before the drop, which are consistent with Hertzian elastic contact solution. However, the large fluctuations in hardness associated with 1 nm indenter indicates its deviation because Hertz’s analysis is valid only in the limit of d ≪ R. The hardness drops here are more pronounced than those in force-depth curves. After the drop, the hardness fluctuates roughly around a constant value, displaying noise that resembles bursts observed in generic nanomechanical studies [33,34]. As to indenter with 7.5 nm radius, its measured hardness is around 12 GPa, which agrees quite well with the previous simulation results [18]. More importantly, an increase in measured hardness corresponds to the decreasing indenter radius, which is consistent with the conventional indentation size effect. As shown in Figure 3b, the pop-in load increases and the maximum shear stress decreases with increasing indenter radius. This is also referred to as the second type of indentation size effect [3], as mentioned above. It is not surprising that measurements based on maximum shear stress are consistent with those in terms of hardness.

Compared with that of the fuzzy signal in the force-depth curves, the energy-depth curves have less fluctuation, as shown in Figure 2b. As mentioned in Ref. [10], the onset of plasticity for both the (111) and (110) orientations can be seen as a sudden drop in load, while pop-in in the (100) orientations is more gradual, and sometimes is unobservable. In other words, dislocation nucleation can be viewed as a minor event, which is almost unobservable in the load-depth curves, before the major “pop-in” event. As explained in Ref. [18], the slip system on (100) surface are more easily activated, since the corresponding Schmid factor is 0.41 (while on (111) surface, it is 0.27). Also, the (111) surfaces are mechanically stiffer so that larger plastic displacement jump would be observed when a considerable elastic energy builds up before dislocation avalanche happens. This confirms the claim by Ref. [35] that the indenter force is not a reliable indicator of the onset of plasticity. To identify the true incipient plasticity under nanoindentation, atomistic observations are required. In the following studies, the force drop will be identified by the associated energy drop combined with tracking of atomic events by visualization.

### 3.2. Uniaxial Tensile Loading

**Figure 4:**
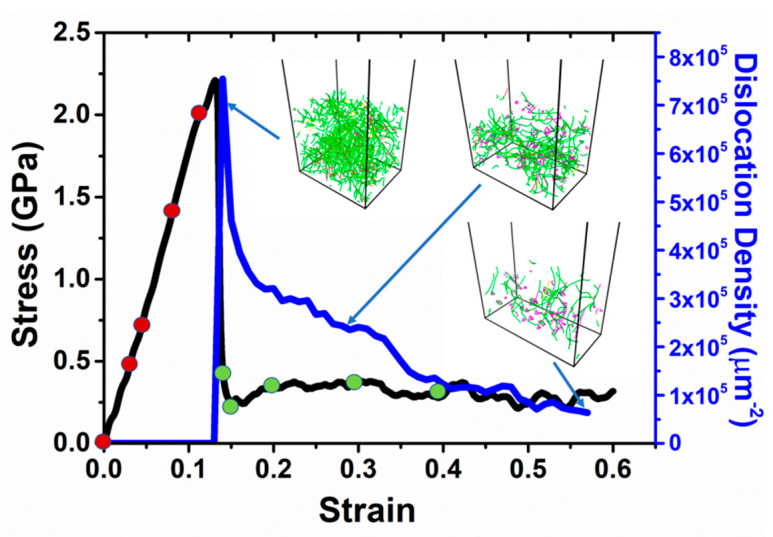
Tensile stress-strain curve (black) and corresponding dislocation density (blue) during the uniaxial loading along x direction. Snapshots of dislocation distribution in substrate shown in inset figures. Red and green cycles represent samples with elastic and plastic deformation, respectively, where the subsequent nanoindentions are conducted after thermal equilibration. Blue, green, pink, yellow, cyan, and red lines represent perfect, Shockley partial, stair-rod, Hirth, Frank, and other types of dislocations, respectively. The color scheme is consistent throughout this work.

Upon completion of 2 ns thermal equilibration process, the uniaxial tensile deformation is carried out at a constant engineering strain rate of 10^9^ s^−1^ along x axis ([100] direction). The stress-strain curve is shown in Figure 4. This simulation is similar to the previous study on deformation behavior in <100> Cu nanowires [36]. Initially, the sample undergoes elastic deformation along x direction with a linear increase in stress up to a peak value followed by a sudden drop. The flow stress fluctuates about 0.25 GPa without significant strain hardening. By detecting the formation and movement of dislocations, we find that the evolution of dislocation distribution is correlated with the observed stress-strain curve. As can be seen in the top-left inset figure, dislocation avalanche occurs when yielding, i.e., drastic drop in stress value is ascribed to nucleation of partial dislocation in an initially defect-free single crystal (in Cu, because of the low stacking fault energy, the partial dislocations dominate). The rapid decrease in dislocation density (dislocation depletion) with sharp decay of stress is believed to be ascribed to the fast movement of the dislocations to the sample free surfaces and annihilations. The lack of dislocation multiplication mechanism at such small length scale leads to dislocation starvation [37,38] because there is no chance that Frank–Read source would be activated due to its reduced dimensions.

### 3.3. Indentations on the Samples with Elastic Deformation

**Figure 5:**
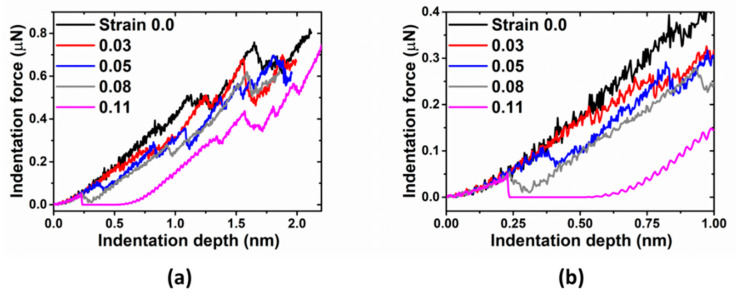
(**a**) Indentation force vs. indentation depth for Cu with elastic deformation, indented with tip radius R = 7.5 nm. (**b**) Indentation force in early stage of indentation.

**Figure 6:**
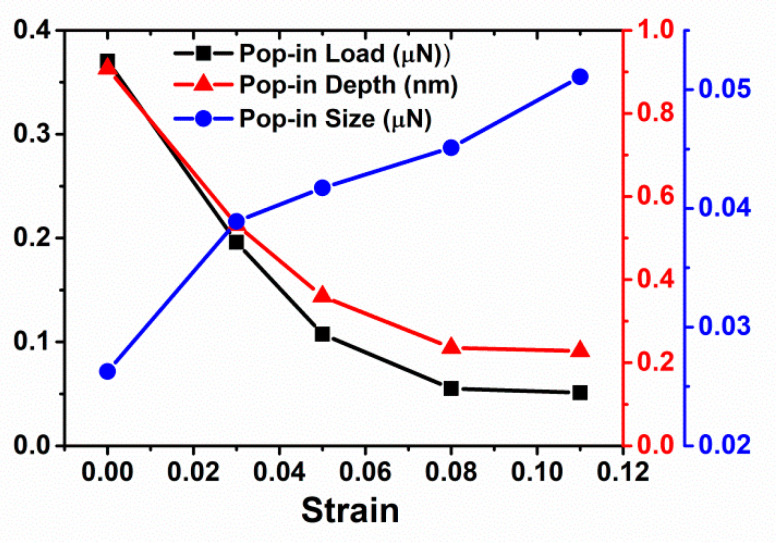
Pop-in load, depth, and size vs. prestrain for Cu with elastic deformation, indented with tip radius R = 7.5 nm.

Before indentation, the sample was held at a constant strain for 500 ps to approach thermal equilibrium state, which is followed by subsequent nanoindentation (see in Figure 5). In this scenario, there is no competing between prestrain and existing dislocations, and only pure strain effect comes into play. For a given indenter tip radius (R = 7.5 nm), the key characteristic of the pop-in (or critical) indentation load and depth decreases in general with an increase of the prestrain (see in Figure 6). While the pop-in size (the magnitude of force drop) generally increased with the prestrain. For strain = 0.11, which are close to the yield point, the indentation force could drop abruptly to zero after elastic deformation. In the elastic range, the nucleation of partial dislocation is much easier in the more prestrained sample due to lower activation energy and bigger activation volume without the presence of pre-existing dislocations.

### 3.4. Indentations on the Samples with Plastic Deformation

**Figure 7:**
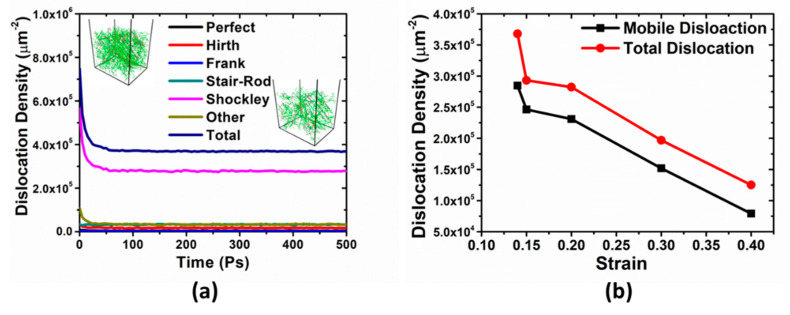
(**a**) Evolution of different types of dislocations during 500 ps equilibrium run before nanoindentations for prestrain 0.14. (**b**) Total and mobile dislocation density vs. prestrain.

Before indentation, the samples were held at constant strains for 500 ps to approach thermal equilibrium state. Figure 7a shows typical curves for evolution of different types of dislocations during such equilibrium process. The dislocation density decays very quickly to a plateau region. The initial and final snapshots of dislocation distribution in the sample are shown in insets in Figure 7a. The abovementioned dislocation starvation also occurs during the equilibrium process. Figure 7b shows the equilibrium total and mobile dislocation density as a function of prestrain. As the prestrain increases, both of the dislocation densities decrease.

**Figure 8:**
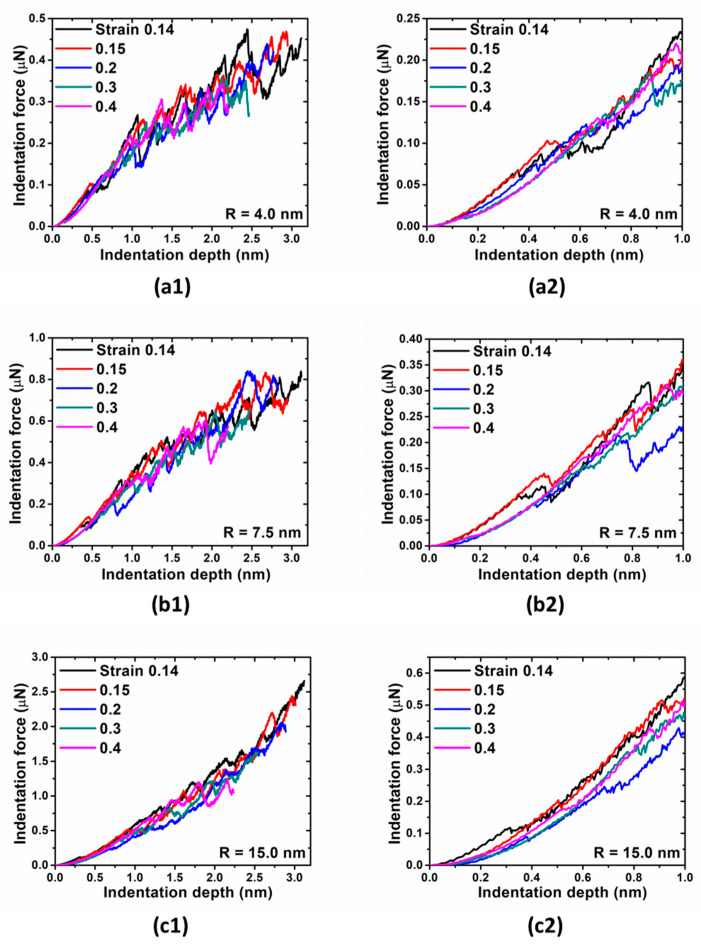
Indentation force vs. indentation depth for Cu under various plastic strain, indented with tip radius (**a1**,**a2**) R = 4.0 nm, (**b1**,**b2**) R = 7.5 nm, and (**c1**,**c2**) R = 15.0 nm, respectively. Indentation force in early stage of indentation is shown in second column.

**Figure 9:**
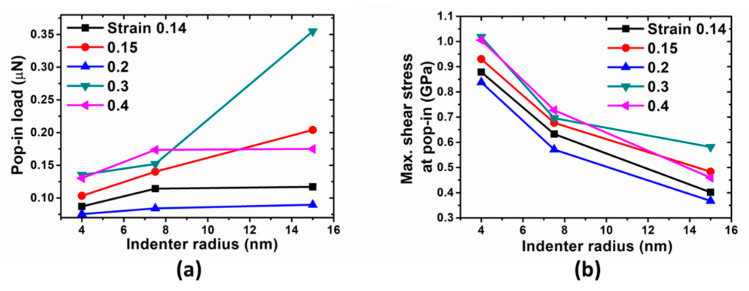
(**a**) Pop-in loads and (**b**) corresponding maximum shear stresses as a function of indenter radius with various strain levels.

In the next step, nanoindentation simulations are conducted. The indentation force-depth behaviors associated with different tip sizes are shown in Figure 8. Even initial deformations are not overlapped, which were observed in previous study [12]. The pop-in loads and corresponding maximum shear stresses as a function of indenter radius with various strain levels are shown in Figure 9. Compared with that of indentations on the samples with the elastic deformation, there is no distinct trend in this case. Especially for strain levels 0.2, the corresponding pop-in loads and maximum shear stresses are the lowest compared with that of other strain levels.

**Figure 10:**
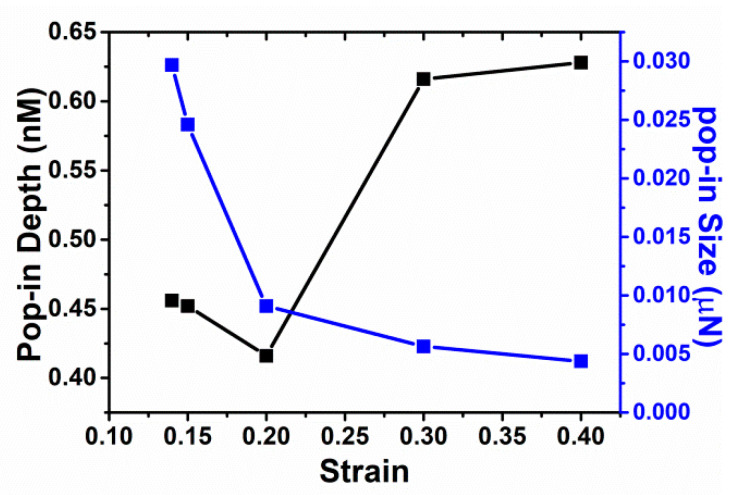
Pop-in depth and size vs. prestrain for Cu with plastic deformation, indented with tip radius R = 7.5 nm.

**Figure 11:**
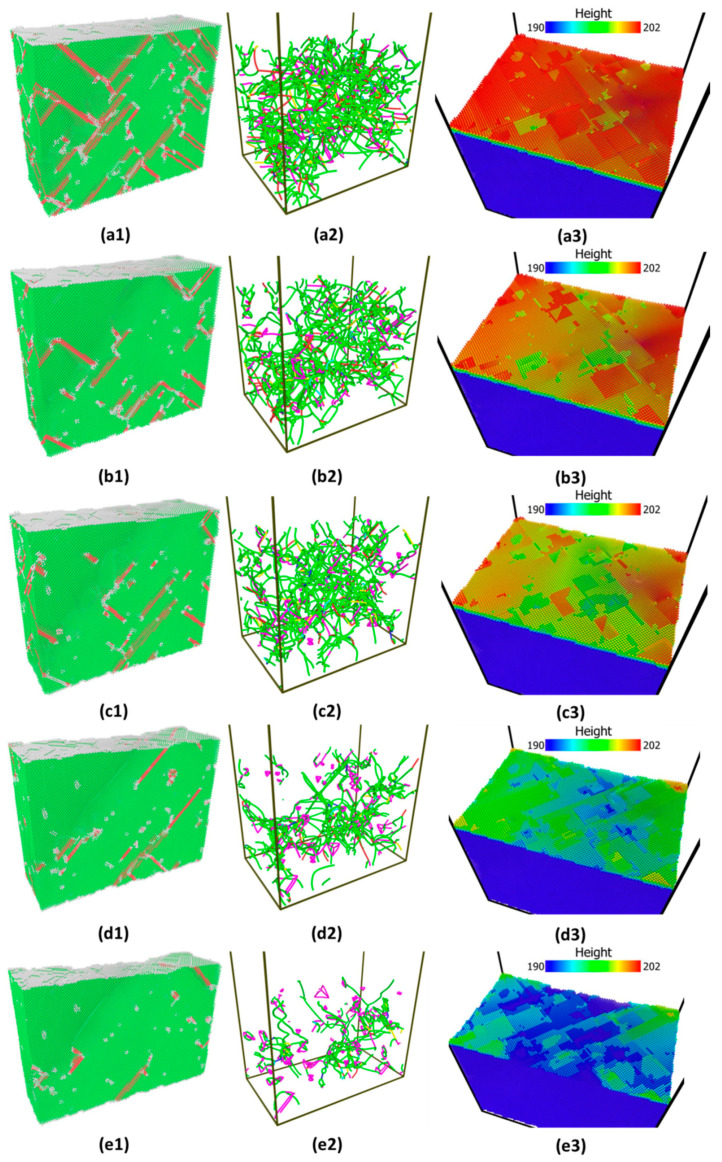
Snapshots of initial atomic structure, distributions of dislocations, and surface morphology before indentations at various strain levels (**a1**–**a3**) 0.14, (**b1**–**b3**) 0.15, (**c1**–**c3**) 0.2, (**d1**–**d3**) 0.3, and (**e1**–**e3**) 0.4.

Let us take the sample indented with tip radius R = 7.5 nm as a reprehensive examples. The variations of pop-in depth and size with the applied strain are also shown in Figure 10, in which the pop-in size decreases with an increase of the prestrain, while the pop-in depth in general increases with the prestrain except strain level 0.2. Based on the atomic animations, we observe that these curves heavily depend on both the distribution of existing dislocation under the indenter and local morphology of substrate surfaces, although the nanoindentation spots (x = 10 nm and y = 10 nm as shown in Figure 1) on the various samples are the same for each prestrain. In other words, the specific distributions of existing dislocations and surface configuration induced by plastic deformation smear out the trend solely due to strain. The distribution of existing dislocation under the indenter and top surface morphology are quite different, as shown in Figure 11. Five indentation simulations indented with tip radius R = 7.5 nm under various prestrains fall into three groups: (1) group 1 including smaller prestrain (0.14 and 0.15), where load drops come from propagation of pre-existing dislocation underneath the indenter tip, which is followed by dislocation nucleation under the tip; (2) group 2 including bigger prestrain (0.3 and 0.4), where load drops result from the nucleation of dislocations from dislocation-free region under the tip while surrounding pre-existing dislocations are not disturbed, and (3) group 3 including prestrain 0.2, where the indenting is close to a surface step.

**Figure 12:**
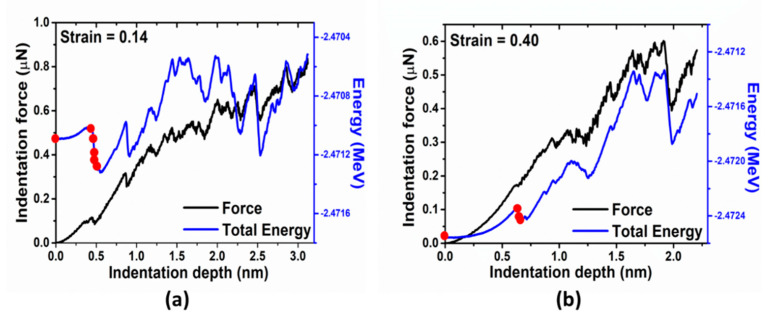
Indentation force and total energy vs. indentation depth for two different prestrain indented with tip radius R = 7.5 nm: (**a**) 0.14 and (**b**) 0.40. Detailed configurations associated with red circles are shown in Figure 13 and Figure 14, respectively.

**Figure 13:**
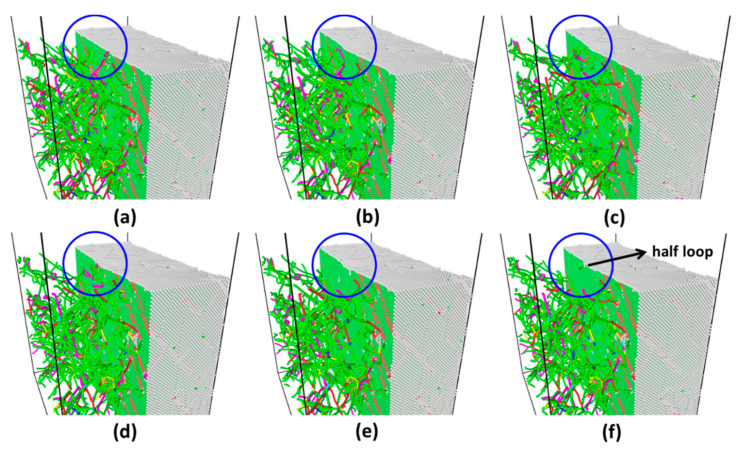
Snapshots of atomic structure and distributions of dislocations across sample under strain 0.14. (**a**–**f**) are corresponding to red circles marked in Figure 12a. Pre-existing dislocations are driven away due to stress field induced by the tip (illustrated by the blue circles). (**e**) Shows a half loop nucleated under tip.

**Figure 14:**
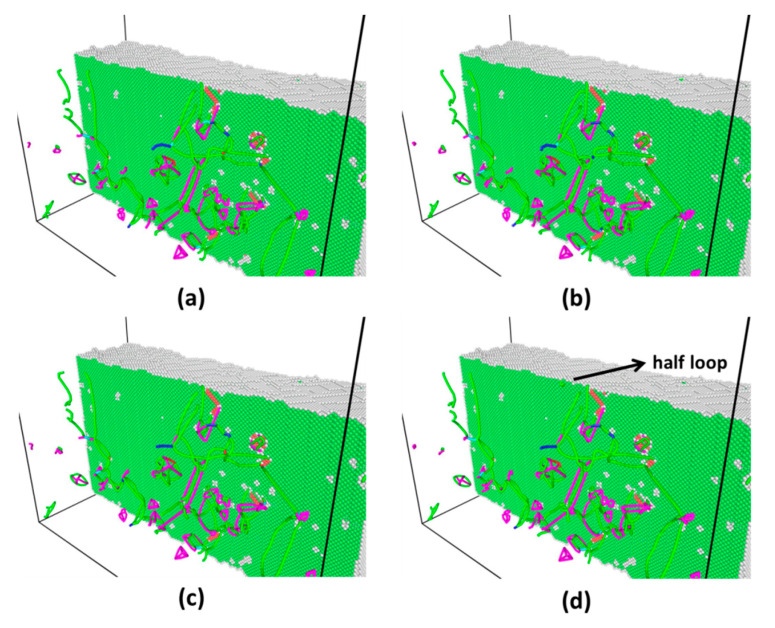
Snapshots of atomic structure and distributions of dislocations across the sample under strain 0.40. **(a**–**d**) are corresponding to red circles marked in Figure 12b. (**d**) Shows a half loop nucleated under the tip.

As the representative instances, the indentation force and total energy versus depth for two different strains (0.14 and 0.40) are shown in Figure 12. Again, energy drop is a better indicator of incipient plasticity than force drop. The evolution of atomic structure and dislocations distributions marked by red cycles in both graphs of Figure 12 can provide detailed explanation for such different behaviors. Figure 13 shows that there exists a network of dislocations distributed across the sample under strain 0.14. As the indentation tip is pressed deeper and deeper (from Figure 13a–e), the pre-existing dislocations are driven away due to the stress field induced by the tip (illustrated by the blue circles), as shown in Figure 13e, where there is no observable dislocation anymore underneath the tip. It is followed by the nucleation of dislocations (a half loop) under the tip (see Figure 13f). As to the strain of 0.40, on the other hand, nucleation of dislocations occurs (Figure 14d) without any effect on the movement of surrounding dislocations. From Figure 14a–d, there are no observable rearrangements of distribution of pre-existing dislocations. 

Plasticity can be initiated either by the activation of pre-existing mobile dislocations (strain = 0.14 and 0.15, see Figure 13) or by the nucleation of dislocations (strain = 0.3 and 0.4, see Figure 14). Obviously, the latter requires higher stresses than the former; this is why we observe that at smaller strain (0.14 and 0.15), critical indentation load and critical indentation depth are smaller than those at the bigger strain (0.3 and 0.4). In the experimental work [4], they found that prestraining measurably reduces the pop-in load due to the increasing initial dislocation density. A similar phenomenon was also found in the discrete dislocation dynamics study [39]. Our current study provides the same physical origin but shows a different trend, which results from decreasing dislocation density with increasing prestrain due to dislocation starvation in the small volume as discussed above.

**Figure 15:**
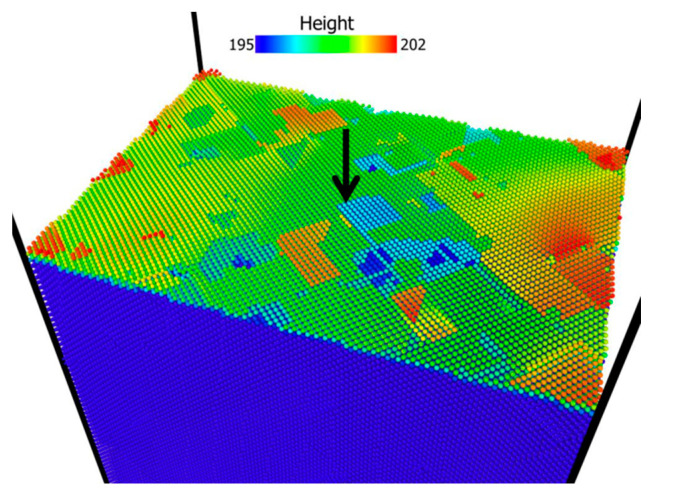
Snapshots of initial surface morphology before indentations at strain level 0.2. Black arrow represents positioning indenter center, which is very close to a surface step.

The question remaining is why pop-in loads and corresponding maximum shear stresses with strain levels 0.2 are the lowest compared with that of other strain levels? It was investigated by J. A. Zimmerman et al. [40] that the load required to nucleate dislocations decreases tremendously when indenting spot is close to the surface step. Figure 15 shows top surface morphology colored by height, in which the indenter center was positioned along black arrow. Clearly, the indenting spot is very close to a surface step, which can explain surprisingly low value of pop-in loads, depth, and the corresponding maximum shear stresses.

The indentation force curve heavily depends on where the indenter is located because both the distribution of existing dislocation and local morphology of substrate surfaces under the indenter is space-dependent. If a large number of indentation simulations can be done in many different spots and then the statistical analysis can be performed, the more precise relationship between pop-in loads and dislocation density can be established. This will be an open question and deserved to be investigated in the future studies.

### 3.5. Reloading and Indentations on the Samples with Elastic Deformation

**Figure 16:**
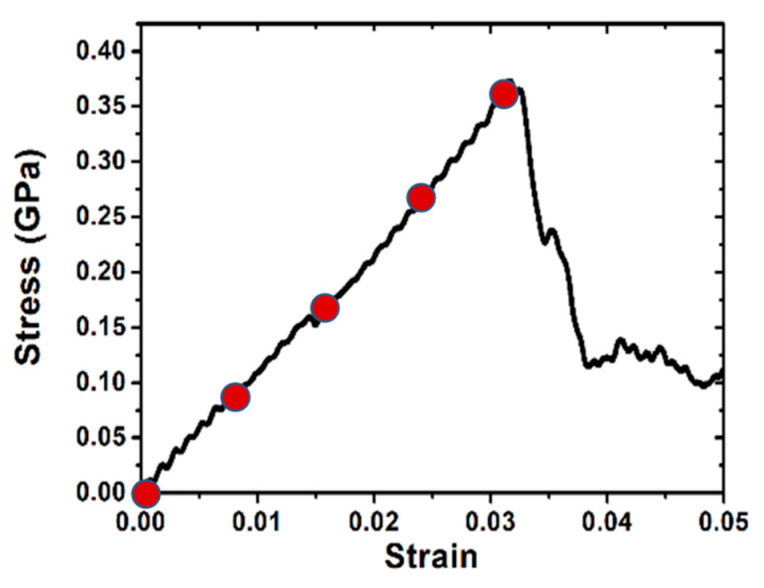
Stress-strain curves in reloading process.

To further investigate the competing effect between prestrain and existing dislocations, we relax and unload the sample to zero stress from strain = 0.6, then reload the sampling to locate yield point. The stress-strain behavior is shown in Figure 16. Finally, the systems in such strain state as a certain percentage of yield stress are identified. Various prestrain values are 0% (no prestrain), 25%, 50%, 75%, and 100% of the strain associated with yield stress. The subsequent nanoindentions are conducted with tip radius R = 7.5 nm on various strain level after thermal equilibration.

**Figure 17:**
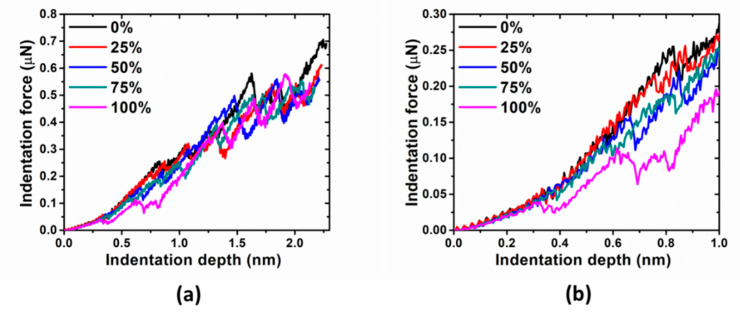
(**a**) Indentation force vs. indentation depth with different prestrains for Cu sample under reloading. (**b**) Indentation force in early stage of indentation.

**Figure 18:**
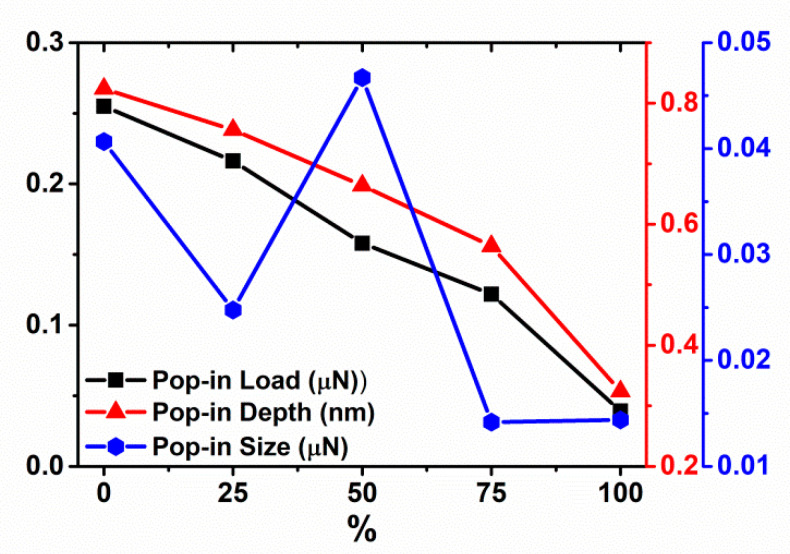
Pop-in load, depth and size vs. prestrain for Cu sample under reloading, indented with tip radius R = 7.5 nm.

The indentation force-depth behaviors associated with different prestrain are shown in Figure 17. The trend in pure elastic strain (as in Figure 6) recovers, i.e., increasing the prestrain results in a decrease in the pop-in load and depth. But the pop-in size has no systematic variation (see in Figure 18).

**Figure 19:**
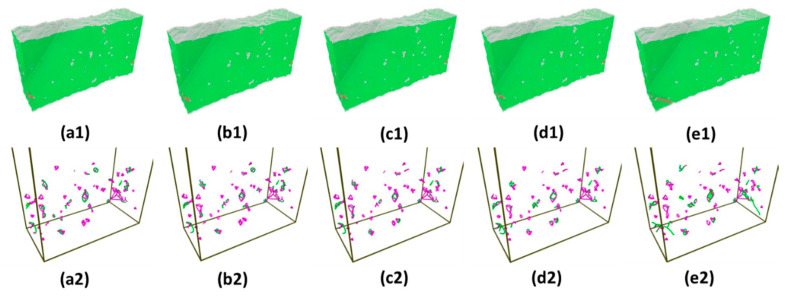
Snapshots of initial atomic structures and distributions of dislocations before indentations at various strain level (**a1**,**a2**) 0%, (**b1**,**b2**) 25%, (**c1**,**c2**) 50%, (**d1**,**d2**) 75%, and (**e1**,**e2**) 100%.

Based on the atomic structures and distributions of dislocations as shown in Figure 19, the dominating dislocation type is stair-rod, which is relatively immobile under stress. Furthermore, there is no much difference in the amount of mobile dislocation such as Shockley partial dislocations in various strain level. In this scenario, the strain effect would overshadow the effect of preexisting dislocations, which resemble case of the samples with elastic deformation in Section 3.3.

## 4. Conclusions

Through large scale MD simulations, nanoindentation of fcc single crystal Cu by a spherical indenter are performed to explore the coupled effects of prestrain, the presence of resulting dislocations and surface morphology, and indenter size on the mechanical response in Cu substrate. We find that two types of indentation size effects are reproduced in defect-free crystals and the trends of indentation hardness and maximum shear stress are consistent. During the indentation process, the total energy change is more sensitive to plastic deformation than the load change, implying that the indenter force is not a reliable indicator of the onset of plasticity. Instead, the total energy of the system is a better indicator. Moreover, we find that in the elastic range, the nucleation of partial dislocations is much easier in prestrained samples due to a lower activation energy and larger activation volume without the presence of pre-existing dislocations. Plasticity can be initiated either by the activation of pre-existing mobile dislocations (prestrain = 0.14 and 0.15) or by the nucleation of dislocations (prestrain = 0.3 and 0.4). The latter requires higher stresses than the former. So, we observe that at smaller prestrains (0.14 and 0.15) pop-in loads and the corresponding maximum shear stresses are smaller than that of the bigger prestrain (0.3 and 0.4). A key finding, related to experimental studies, is that the load needed to nucleate dislocations decreases tremendously when indenting spot is close to the surface step, which can explain surprisingly low value of pop-in loads and corresponding maximum shear stresses at prestrain 0.2. Finally, it is worth noting that after unloading and reloading to a new yield point, subsequent nanoindentions conducted on various strain levels show that the strain effect would overshadow the effect of pre-existing immobile dislocations (stair-rod type), resembling a quasi-elastic response.

## Data Availability

The data presented in this study are available on request from the corresponding author. The data are not publicly available due to privacy reasons.
